# Occluded-Object 3D Reconstruction Using Camera Array Synthetic Aperture Imaging

**DOI:** 10.3390/s19030607

**Published:** 2019-01-31

**Authors:** Zhao Pei, Yawen Li, Miao Ma, Jun Li, Chengcai Leng, Xiaoqiang Zhang, Yanning Zhang

**Affiliations:** 1 Key Laboratory of Modern Teaching Technology, Ministry of Education, Xi’an 710119, China; zpei@snnu.edu.cn; 2 School of Computer Science, Shaanxi Normal University, Xi’an 710119, China; liyawen@snnu.edu.cn (Y.L.); mmthp@snnu.edu.cn (M.M.); 3 School of Computer Science, Nanjing Normal University, Nanjing 210046, China; lijun_enbo@126.com; 4 School of Automation, Southeast University, Nanjing 210096, China; 5 School of Mathematics, Northwest University, Xi’an 710127, China; ccleng@nwu.edu.cn; 6 School of Information Engineering, Southwest University of Science and Technology, Mianyang 621010, China; xqzhang@swust.edu.cn; 7 School of Computer Science, Northwestern Polytechnical University, Xi’an 710129, China

**Keywords:** synthetic aperture imaging, occluded-object 3D reconstruction

## Abstract

With the three-dimensional (3D) coordinates of objects captured by a sequence of images taken in different views, object reconstruction is a technique which aims to recover the shape and appearance information of objects. Although great progress in object reconstruction has been made over the past few years, object reconstruction in occlusion situations remains a challenging problem. In this paper, we propose a novel method to reconstruct occluded objects based on synthetic aperture imaging. Unlike most existing methods, which either assume that there is no occlusion in the scene or remove the occlusion from the reconstructed result, our method uses the characteristics of synthetic aperture imaging that can effectively reduce the influence of occlusion to reconstruct the scene with occlusion. The proposed method labels occlusion pixels according to variance and reconstructs the 3D point cloud based on synthetic aperture imaging. Accuracies of the point cloud are tested by calculating the spatial difference between occlusion and non-occlusion conditions. The experiment results show that the proposed method can handle the occluded situation well and demonstrates a promising performance.

## 1. Introduction

Object reconstruction is the process of recovering 3D information from a sequence of 2D images. All images in the sequence captured from different perspectives contain the same target object. Three-dimensional reconstruction from image sequences is an important task in computer vision in numerous applications, such as virtual reality, medical-tissue modeling, cultural-heritage conservation, and 3D printing. Despite its extensive applications, the development of object reconstruction still has plenty of challenges. Facilitated by powerful hardware-computing capabilities, most of current 3D reconstruction methods benefit from sufficient input images [1,2,3,4,5,6]. Since partial occlusion leads to certain facets of a target object out of sight, conventional 3D reconstruction methods usually suffer from object occlusion with degraded performance, and thus cannot well handle the cases when the occluded object is present.

**Figure 1:**
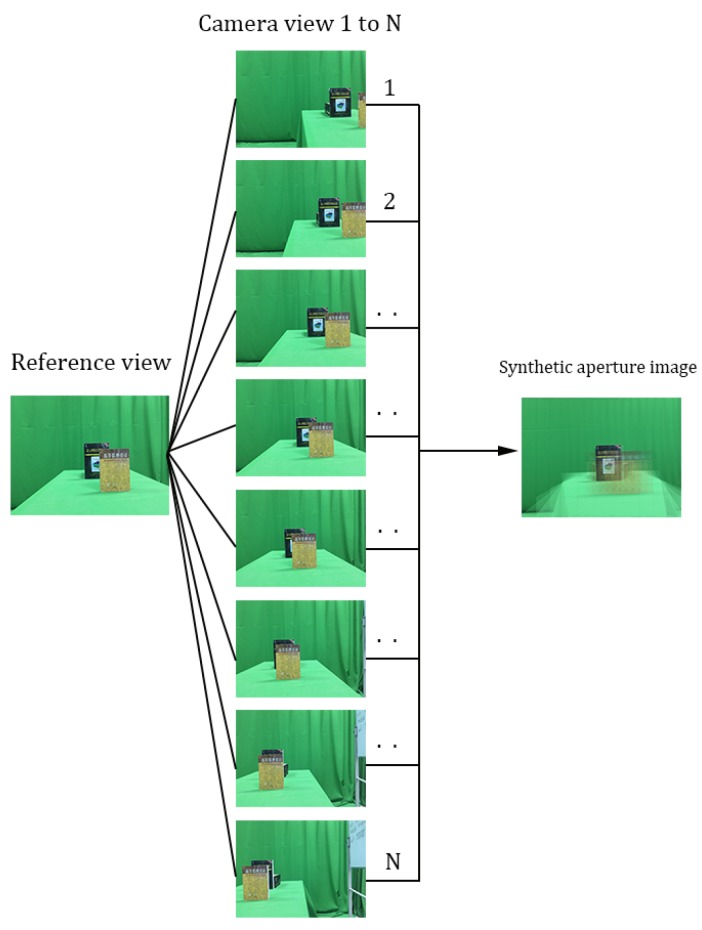
Synthetic aperture imaging.

**Figure 2:**
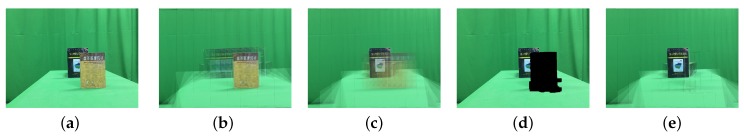
Two books in this scene. (**a**) Reference view. (**b**) Synthetic aperture imaging focused on the front book. (**c**) Synthetic aperture imaging focused on the book behind with the blurry book region. (**d**) Result of removing occlusion via pixel labeling. (**e**) Result of synthetic aperture imaging by removing the front book.

To solve the above problem in 3D reconstruction, in this paper, we propose a novel method based on synthetic aperture imaging with pixel labeling. Synthetic aperture imaging (SAI) is a technique that mimics a large virtual convex lens achieving digital refocusing. More specifically, an array of cameras is used to capture different images that are subsequently leveraged for synthesizing images in different depths (Figure 1). According to the distance between the object and camera plane, all the images can be warped into the depth of the occluded object for generating synthetic aperture images [7,8]. Thus, one can ‘see through’ the occlusion and focus on the occluded objects. Taking advantage of the above assumption, a method for detecting occluded objects was proposed in References [9,10]. In synthetic aperture images, the part of the object located at the focal plane is sharp, while the other part is blurry (Figure 2b,c). Motivated by the above observation, we use a camera array as a depth sensor for 3D reconstruction, and propose a method which applies SAI to handle the occlusion problem in 3D reconstruction, showing its promise in experimental evaluations.

In this paper, we propose a novel method to reconstruct occluded objects based on SAI. Based on the characteristics of SAI, the proposed method can be applied in many areas with occlusion, such as occluded-object reconstruction in indoor and outdoor scene, rock climbing routes reconstruction [11], etc. Depending on the number of camera views in the camera array, the proposed algorithm only requires a few images involved. A clear image of the occluded object can be obtained by using SAI, and the 3D reconstruction of the occluded object is achieved by combining the corresponding distance information. To calibrate the above-mentioned cameras, we employ the off-the-shelf calibration model in open-source software OpenCV to obtain the camera’s internal parameters. The relative-position relationship between cameras is calculated by using the principle of triangulation [7], which provides the basic condition for SAI. Please note that the virtual array of a sliding camera is used in our experiment to improve the system flexibility and adapt to a significantly varying environment.

The main contributions of this paper are summarized as follows. We have proposed a novel method to reconstruct an occluded object in 3D based on SAI. Compared with conventional/traditional 3D reconstruction methods based on feature extraction and matching, our method only requires the distance between object and camera array without costly feature extraction and matching involved extraction and matching feature.

The rest of this paper is organized as follows. After reviewing related works in Section 2, the proposed method is introduced in Section 3. Then, experiment results are presented in Section 4. In Section 5, we quantitatively evaluate the proposed method and conduct extensive comparative studies. Finally, a conclusion is drawn in Section 6.

## 2. Related Works

In the field of multiview object reconstruction, massive and numerous methods have been proposed in recent years. Qian et al. [12] presented the first approach for simultaneously recovering the 3D shape of both a wavy water surface and a moving underwater scene. Xu et al. [13] captured the inherent geometrical variation of underwater objects for reconstruction, requiring sufficient corresponding features among input images. Ebner et al. [14] proposed a fully automated algorithm for the 3D reconstruction of real objects, achieving a high-quality point cloud by exploiting pairwise stereo depth estimation. Shen [15] merged a depth map with a patch-based stereo matching process for reconstructing objects in large-scale scenes. Whiting et al. [11] used multiview stereo and the reference videos of a climber in action to reconstruct the rock wall.

During the past few decades, tremendous progress has been made in research on Structure from Motion (SFM), which plays an important role in 3D reconstruction, Simultaneous Localization and Mapping (SLAM), Virtual Reality (VR), Augmented Reality (AR), and other fields. The earliest relevant research originates from building self-calibration metric reconstruction systems based on the framework in References [16,17,18,19,20]. Built on these systems, the first system of unordered Internet photo collections [21,22] and urban scenes [23] was devised. Due to the continuous improvement of computing performance, large-scale reconstruction systems have been developed for handling hundreds of thousands [1], millions [2,3,4,5], and even a hundred million Internet photos [6]. The incremental strategy for SFM has emerged as mainstream research, and become the most commonly used 3D reconstruction approach at present [1,2,3,22,24].

Many camera array systems have been built over recent decades, like the Stanford multicamera array. Their device was designed to record a synchronized video dataset from over a hundred cameras to a hard-disk array [25]. Zhang and Chen developed a self-reconfigurable camera array system [26] to capture video sequences from an array of mobile cameras, dynamically render novel views, and reconfigure camera positions to achieve better rendering quality. Joshi et al. [27] presented a method to track a 3D object through significant occlusion using multiple nearby cameras. Fujii et al. [28] built a multipoint-measuring system which was more than 100 points in a “synchronized” manner. Based on the University of Alberta camera array system, Lei et al. presented a new approach to recovering spatially and temporally consistent depth maps from multiple video sequences [29], while Pei et al. presented a novel multiobject detection method in complex scenes [9]. In summary, these camera array systems can be classified into many categories, such as high-speed videography systems [30], high-performance imaging systems [31] and image-based rendering systems [29]. Gasparovic and Gajski proposed a two-step process for calibrating ultrawide-angle cameras [32]. Based on the camera-calibration method using images taken from an unmanned aerial vehicle [33], Perez et al. [34] proposed a low-cost surveying method using unmanned aerial vehicles. Vaish et al. [7,8] proposed an efficient method to calibrate a dense camera array using plane and parallax, making SAI a powerful tool. Do et al. [35] made use of independent component analysis and K-means method to reconstruct occluded objects with a camera array. Pei et al. [36,37] proposed a labeling method to separate a target object from background, and showed superior performance surpassing the traditional methods in reducing the effect of moderately or heavily occluded objects. In addition, Pei et al. [38] proposed a method to evaluate the image quality of SAI.

**Figure 3:**
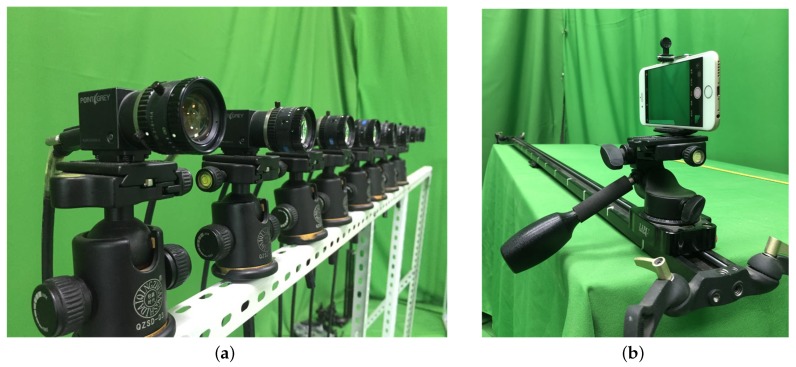
(**a**) Camera array. (**b**) Camera slide rail.

Despite their success, the above-mentioned 3D reconstruction methods cannot effectively reconstruct a target object in the case when a limited number of images are involved. Besides, traditional 3D reconstruction methods either assume occlusion is absent from the scene or remove the occlusion from the reconstructed result in a straightforward manner, and thus can only handle the occlusion-free situations [39,40]. Based on SAI, our proposed method uses pixel labeling to reconstruct a 3D object and achieves satisfactory 3D reconstructed results as shown in Figure A2. In addition, traditional camera arrays can handle multiple scenes yet move inconveniently. Too complicated transmission lines and dependence on a power supply make it difficult for them to be used in outdoor scenes. To address the limitation, we use the camera array with a slide rail to simulate a single-row camera array in space and time, which greatly enhances its flexibility to adapt to more varying environments, as shown in Figure 3b.

## 3. SAI-Based Reconstruction Method

**Figure 4:**
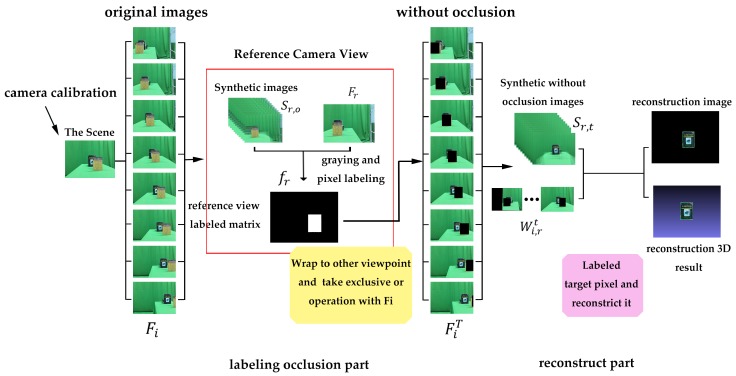
The processing pipeline of the proposed algorithm.

With the help of SAI, the occluded object can be reconstructed with the following steps. To begin with, the camera needs to be calibrated to obtain its internal parameters. Meanwhile, the relative-position relationship between cameras is calculated by using the principle of triangulation, which provides the basic condition for SAI. Then, the SAI method which plays a key role in the entire algorithm is presented, running through the subsequent steps. Next, occluded pixels are labeled based on SAI, and image matting is introduced, which corresponds to the labeling-occlusion part shown in Figure 4. Finally, the reconstruction method without occlusion is presented, which corresponds to the reconstructed part shown in Figure 4.

### 3.1. Camera Array Calibration

A camera array is constructed by placing multiple cameras parallel to each other on a shelf. It enables capturing images from different perspectives at the same time. By adjusting the exposure time, aperture size, and other parameters of each camera, more information can be obtained than single camera. In our method, we use the relationship between plane and parallax to refocus the captured image, providing a technical basis for the following procedures. First, to eliminate the effect of distortion on subsequent operations, we exploit the off-the-shelf calibration model in OpenCV library available publicly to calibrate each camera with about 12 images. More specifically, the calibration module uses pinhole model of the camera and the Brown’s lens distortion model to generate the undistorted image coordinates represented by:

(1) $$x_{coor}=x\left(1+k_{1}R^{2}+k_{2}R^{4}+k_{3}R^{6}\right)+\left[2b_{1}xy+b_{2}\left(R^{2}+2x^{2}\right)\right],$$

(2) $$y_{coor}=y\left(1+k_{1}R^{2}+k_{2}R^{4}+k_{3}R^{6}\right)+\left[b_{1}\left(R^{2}+2y^{2}\right)+2b_{2}xy\right],$$

where *x* and *y* are reduced image coordinates, while *R* is square radial distance. Thus, we can obtain the distortion parameters, the radial distortion parameters ($k_{1}$, $k_{2}$, $k_{3}$) and the tangential distortion parameters ($b_{1}$, $b_{2}$).

**Figure 5:**
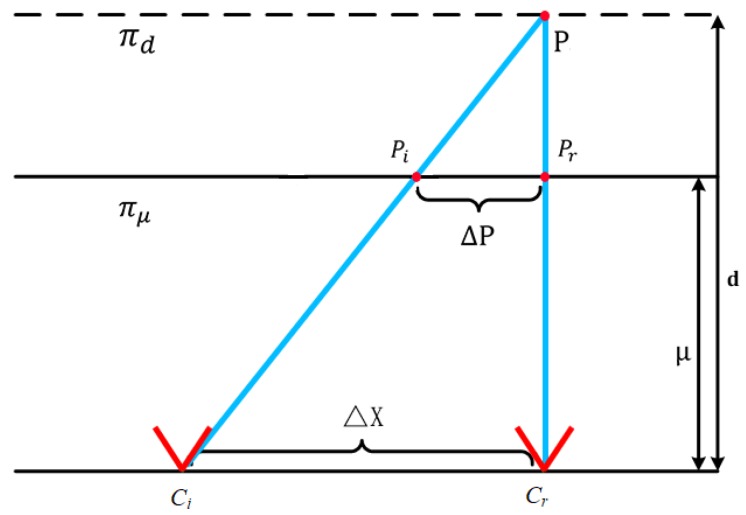
The planar parallax for camera array.

Then, the camera array is calibrated with the method [7]. After fixing the camera array, the calibration board is used to take multiple sets of data for calibrating the camera array. In particular, the calibration board remains parallel to the camera array plane during capturing. Next, one set of data is selected as the reference plane $\pi_{\mu }$, and the distance between this set of data and the camera array is called the reference distance μ. Then, one of the cameras in the camera array is selected as the reference camera $C_{r}$. The homography matrix $H_{i,r}$ from different cameras to the reference camera is calculated according to the corner points, and will be applied to the following steps. As can be seen from Figure 5, a point *P* beyond the reference plane has distinct images $p_{i}$, $p_{r}$ in cameras $C_{i}$, $C_{r}$. The parallax between these two images depends on the relative camera displacement ΔX and relative depth $\frac{\left(d-\mu \right)}{d}$, which can be formulated as:

(3) Δp=ΔX·a.

Multiple parallaxes can be obtained by capturing multiple sets of calibration boards at different depths, resulting in the camera displacement ΔX. In practice, we use SVD to obtain the closest result. Calibration results are used for subsequent operations.

### 3.2. Synthetic Aperture Imaging

A synthetic aperture image on an arbitrary focal plane that is parallel to the camera plane can easily be computed according to the parallax and parallel synthetic method [7]. With N cameras in the camera array, each camera is denoted by $C_{i}$(i∈[1,N]) while $F_{i}$ is denoted as a frame captured by camera $C_{i}$. Among these N cameras, a camera is chosen as reference camera $C_{r}$, r∈[1,N]. Without loss of generality, we choose the center position as reference camera. The homography matrix $H_{i,r}$ which is generated from camera array calibration is used to warp $F_{i}$ to reference camera $C_{r}$ on reference plane $\pi_{\mu }$ by:

(4) $$W_{i,r}^{\mu }=H_{i,r}\cdot F_{i},$$

where i=1,⋯,N and $W_{i,r}^{\mu }$ denotes the warped image from frame $F_{i}$ to frame $F_{r}$ after homography transformation. Since the homography matrix is obtained in the reference plane, μ represents the image projected onto the reference plane. The relative positions between cameras are represented by displacement matrix ΔX that can be generated from the calibration results of the camera array. We denote $\pi_{d}$ as the target focal plane at depth *d* within a depth range, and $\pi_{d}$ is parallel to reference plane $\pi_{\mu }$. As shown in Figure 5, we can easily calculate the ratio of the relative depths between $\pi_{d}$ and $\pi_{\mu }$ by:

(5) $$a=\frac{\left(d-\mu \right)}{d}.$$

According to the method in Section 3.1 [7], the parallax matrix Δp at depth *d* can be obtained by Equation (3). Using the parallax matrix Δp, we focus on target plane $\pi_{d}$ by translating images $W_{i,r}^{\mu }$. Specifically, we select a depth range covering the occlusion and the occluded object. Thus, image $W_{i,r}^{\mu }$ is shifted by Δp using:

(6) $$W_{i,r}^{d}=\left[\begin{array}{cc} I & \Delta p\\ \theta & 1 \end{array}\right]\cdot W_{i,r}^{\mu },$$

where $W_{i,r}^{d}$ denotes the shifted image focusing on depth *d*. I is a 2×2 identity matrix. θ is a two-dimensional zero vector. $W_{i,r}^{d}\left(q\right)$ is denoted as the value of pixel *q* in the image. Finally, $S_{r,d}\left(q\right)$ is the value of pixel *q* in the corresponding synthetic aperture images. We have:

(7) $$S_{r,d}\left(q\right)=\frac{1}{N}\sum_{i=1}^{N}W_{i,r}^{d}\left(q\right).$$

With Equation (7), synthetic aperture image $S_{r,d}$ focusing on depth *d* is generated by averaging pixel values in all warped images $W_{i,r}^{d}$. Intuitively, the synthetic images are illustrated in Figure 2b,c.

To summarize, we first project the images of different camera views onto the reference view $W_{i,r}^{\mu }$ according to the homography matrix obtained by Equation (4). Next, with the relative depths from the camera array plane and the relative camera displacement obtained by camera calibration, the parallax Δp between images $F_{i}$ and $F_{r}$ at depth *d* is calculated by using Equations (3) and (5). Then, according to Equation (6), $W_{i,r}^{d}$ is obtained by transforming different views into the corresponding depth of the reference view. Finally, the synthetic aperture image $S_{r,d}$ is obtained by averaging the sum of the pixel values of all $W_{i,r}^{d}$. SAI running through the subsequent steps serves as the cornerstone in the entire algorithm.

### 3.3. Labeling of Occluded-Region Pixels Using Synthetic Aperture Imaging and Image Matting

As shown in the labeling-occlusion part of Figure 4, we obtain a set of synthetic aperture images focused on the depth range of the occlusion before labeling an occluded region. The key idea of removing the occlusion is to find and label its pixel area. Please note that images related to the labeling process are converted into gray intensity to reduce the effect of illumination and color on reconstruction results.

With the starting and ending depth of the occlusion location denoted as α and β, respectively, synthetic images $S_{r,o}$ that focus on depth *o* can be generated from the above-mentioned operation. In addition, $F_{r}$ denotes the image of the reference camera view, while $F_{r}\left(q\right)$ represents the pixel value of point *q* in images. After graying all pixels in $S_{r,o}$ and $F_{r}$, respectively, we can obtain grayscale $S_{r,o}^{g}$ and $F_{r}^{g}$. Similarly, $S_{r,o}^{g}\left(q\right)$ and $F_{r}^{g}\left(q\right)$ are the pixel values of the point *q* in grayscale. The pixels of the occlusion in these images can be distinguished by variance:

(8) $$var\left(q\right)={\left(F_{r}^{g}\left(q\right)-S_{r,o}^{g}\left(q\right)\right)}^{2},$$

where $var\left(q\right)$ is the difference of pixel *q* between reference camera-view image $F_{r}^{g}$ and synthetic image $S_{r,o}^{g}$. In the synthetic aperture image $S_{r,o}^{g}$, the same depth points will be focused and close to the correspond point in reference image $F_{r}^{g}$. Depending on the value of the variance, a threshold is empirically predefined to select these pixels. To be specific, pixel *q* in synthetic aperture images $S_{r,o}$ is labeled by:

(9) $$f_{r,o}\left(q\right)=\left\{\begin{array}{cc} 1 & \mathrm{if}\;var\left(q\right)\leq \delta \\ 0 & \mathrm{otherwise} \end{array}\right..$$

At the depth of *o*, $f_{r,o}\left(q\right)$ is defined as the label value of point *q* in reference camera view $F_{r}$. δ is denoted as the threshold of variance and can be adjusted according to the result of $f_{r,o}$ indicating the labeled matrix at depth *o* to obtain the best result. For example, when synthetic aperture image is focused at depth *o* with the pixel on the object, variance for the corresponding points in different views will be small. Otherwise, if the pixel is off the focal plane, all the corresponding points will be at different positions in the synthesis image. Thus, object at that depth is clear and other parts are blurred. With depth *o* valued in the range (α,⋯,β), a label matrix set is derived from Equation (9). Then, label matrix $f_{r}$ of occlusion on the reference camera view is merged with each label matrix $f_{r,o}$. If any label matrix $f_{r,o}$ is 1 at the same point in different depth *o*, the $f_{r}$ at same point position is 1, otherwise, it is 0. In addition, we employ morphological methods like corrosion, expansion, and the method of image matting [41] to make $f_{r}$ fit the occlusion area. After obtaining and labeling the pixels of occlusion in the reference camera view, we transform $f_{r}$ to other camera perspectives as $f_{i}$. According to Equations (4) and (6), an inverse operation is imposed on $f_{r}$:

(10) $$f_{i}^{\mu }={\left[\begin{array}{cc} I & \Delta p\\ \theta & 1 \end{array}\right]}^{-1}\cdot f_{r},$$

and

(11) $$f_{i}={H_{i,r}}^{-1}\cdot f_{i}^{\mu }.$$

The label matrix $f_{i}$ corresponding to the camera view can be obtained by the above operation. Then, we take the exclusive OR operation of $f_{i}$ and $F_{i}$, while the occlusion part is a black block in the original image:

(12) $$F_{i}^{T}=F_{i}⊕f_{i},$$

where $F_{i}^{T}$ represents the image without occlusion, *T* indicates non-occlusion, and ⊕ denotes the exclusive OR operation. For example, if a point in $f_{i}$ is 0, the correspond position in $F_{i}$ will be retained, otherwise it will be removed. Image set $F_{i}^{T}$, (i∈[1,N]) is used as a new input for SAI and 3D reconstruction introduced in the next section.

### 3.4. 3D Reconstruction of Occluded Object

In the previous section, we have obtained occlusion-free images for each perspective $F_{i}^{T}$(i∈[1,N]), as shown in the reconstruction part in Figure 4. In this section, we use the set of images $F_{i}^{T}$(i∈[1,N]) as a new input to the synthetic aperture to derive synthetic images that are focused on the depth of the occluded object. To be specific, it comprises the following steps. Firstly, the pixel of the object is labeled. Secondly, the corresponding *x* and *y* coordinates are translated from the image coordinate system to the world coordinate system. Then, these pixel values are saved, and the values of all points are derived from the synthetic images. Finally, the occluded object is reconstructed according to the above operations.

In addition, we replace $F_{i}$ in Equation (4) with $F_{i}^{T}$. We assume that $\pi_{t}$ is the plane where the occluded object is located, and *t* is the depth range of the object. Thus, the ratio of the relative depths between $\pi_{t}$ and $\pi_{\mu }$ can be calculated by Equation (5). Δp can also be derived from Equation (3), while images $F_{i}^{T}$ are warped from depth μ to depth *t*.

$S_{r,t}$ is denoted as the synthetic images at depth *t* on the reference camera view *r*. $S_{r,t}\left(q\right)$ is the pixel value of point *q* in the corresponding SAI $S_{r,t}$. The values of all shifted images on *N* are averaged by Equation (7) yielding $S_{r,t}\left(q\right)$. The synthetic occlusion-free aperture image $S_{r,t}$ is shown in Figure 2e. With the help of SAI, the removed occlusion region in Figure 2d can be filled. In contrast to Section 3.3, we use SAI to fix the occlusion region instead of labeling it.

Unlike Equation (8), we use $W_{i,r}^{t}$ to extract pixels from the occlusion area and record the minimum value. New variance $var_{t}\left(q\right)$ can be obtained by a variance method with Equation (13).

(13) $$var_{t}\left(q\right)=\min{\left(W_{i,r}^{t}\left(q\right)-S_{r,t}\left(q\right)\right)}^{2},$$

$M_{r,t}$ is an image that only records the pixel value of the occluded object at depth *t*. $M_{r,t}\left(q\right)$ represents the pixel value of point *q* in image $M_{r,t}$. In addition, η is set as the threshold to take the pixels of the occluded object.

(14) $$M_{r,t}\left(q\right)=\left\{\begin{array}{cc} S_{r,t}\left(q\right) & \mathrm{if}\;var_{t}\left(q\right)\leq \eta \\ 0 & \mathrm{otherwise} \end{array}\right..$$

In $M_{r,t}$, the three-channel pixel value of the corresponding point at current depth *t* is saved. The point cloud of the occluded object can be produced by the 3D information including the plane coordinates, the depth of the corresponding coordinates, and the three-channel pixel value. The point cloud is represented by:    

(15) $$P\left(q\right)=\left(X_{q},Y_{q},t_{q},M_{r,t}\left(q\right)\right),$$

where $P\left(q\right)$ denotes the world coordinates of corresponding point *q* and its three-channel pixel value. $t_{q}$ is the depth of point *q*. World coordinates $\left(X_{q},Y_{q}\right)$ are represented by:

(16) $$X_{q}=\frac{\left(x_{q}-u_{0}\right)\cdot t_{q}}{\lambda }$$

and

(17) $$Y_{q}=\frac{\left(y_{q}-v_{0}\right)\cdot t_{q}}{\lambda },$$

respectively. $x_{q}$ and $y_{q}$ are the coordinates of point *q* in image coordinate system, λ is the focal length expressed in pixel units, while $u_{0}$ and $v_{0}$ are the central origin of the image coordinate system, and can be obtained by camera calibration. After producing all 3D information of the occluded object points, the final 3D reconstruction result is produced as shown in Figure A2.

Figure 4 shows the workflow of the 3D reconstruction process. First, it is necessary to calibrate the camera and camera array to obtain its internal and external parameters, resulting in the undistorted images used as input for the next step. Then, we focus on the occluded plane by warping all images to the depth of occlusion, and label the occlusion-region pixels via variance as shown in the labeling-occlusion part in Figure 4, Section 3.3. Afterward, we refocus on the occluded object and generate synthetic aperture images without these labeled pixels. An extraction method based on pixel variance can be used to distinguish focused objects from an unfocused background. Finally, we extract occluded objects in different depths via variance and project the occluded-object points from the image coordinate system to a world coordinate system in combination of corresponding depth. Thus, the occluded object can be reconstructed. The reconstructed part corresponds to Section 3.4, and the pseudocode of the proposed algorithm is shown in Algorithm 1.

**Algorithm 1** 3D reconstruction of occluded object with camera array using synthetic aperture imaging.
Calibrate camera array;capture image sequence of the occluded object;**for** each depth *o* where the occlusion locates **do** Shift every camera-view image $F_{i}$ to reference camera view *r* at depth *o* using Equations (3)–(6); Generate synthetic images focused on depth *o* using Equation (7); Label pixels in each depth *o* using Equations (8) and (9);
**end for**
Merge label matrices of all depths together in reference camera view $C_{r}$**for** each camera view $C_{i},i=1$ to *N* **do** Transform label matrices $f_{r}$ from reference camera view *r* to other views *i* according to Equations (10) and (11); Take the exclusive OR operation of $f_{i}$ and $F_{i}$ in each camera view using Equation (12);
**end for**
**for** each depth *t* in which the occluded object is located **do** Generate synthetic without-occlusion images that focus on depth *t* using Equations (3)–(6); Extract pixels using Equations (13) and (14); Calculate the coordinate value of each pixel, and convert the image coordinate system to the world coordinate system using Equations (16) and (17); Save those pixels’ information to a file with Equation (15);
**end for**



## 4. Experimental Results

In this section, with both indoor and outdoor scenes involved in our experimental evaluations, two types of camera arrays were used to demonstrate the effectiveness of our proposed method. The first camera array (Figure 3a) consisted of eight Point Grey Research Flea2 color cameras, each of which has a resolution of 640 × 480, and their focal lengths were 751.2 measured in pixel units. The distance between two cameras was approximately 13 cm. The second camera array used in our experiments was virtual and used a horizontal slider rail with iPhone 6. The distance between the two viewpoints was roughly 10 cm, while the distance between the starting and the ending points was approximately 80 cm. It can cope with complex experimental conditions well (Figure 3b).

**Figure 6:**
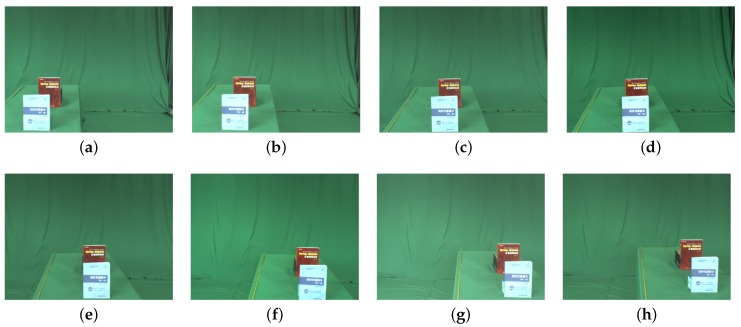
Examples of input book images. (**a**–**h**) are captured images by camera array in the same scene.

The first scene included two books located in a line facing the camera array. We made use of the camera array (Figure 3a) to capture this scene, and the input image sequence consisted of eight images of 640 × 480 pixels. Examples of the input images are shown in Figure 6.

Firstly, we used Equations (3), (4), (6) and (7) to generate the synthetic aperture images focusing on the front book. The region of the front book was clear, and the other region was blurry. Pixels of the front book were labeled with the variance method in Equation (8). We have also obtained the images from which the occlusion was removed. Then, we refocused on the book behind.

**Figure 7:**
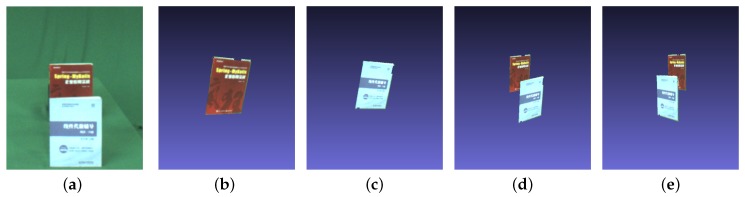
Reconstruction result of the book scene. (**a**) Reference camera view of the book scene. (**b**) Reconstruction result of the book behind. (**c**) Reconstruction result of the front book. (**d**,**e**) Reconstructed book scene observed from different views.

According to Equations (13)–(15), the point cloud was generated based on synthetic aperture images that focus on the book behind. The reconstruction result is shown in Figure 7b. Likewise, the point cloud of the front book was generated in the same way (Figure 7c). Thus, the whole scene containing two books was recovered by combining the two reconstruction results, leading to 24,303 points in this scene. Figure 7 qualitatively illustrates the point cloud presented from different perspectives.

**Figure A1:**
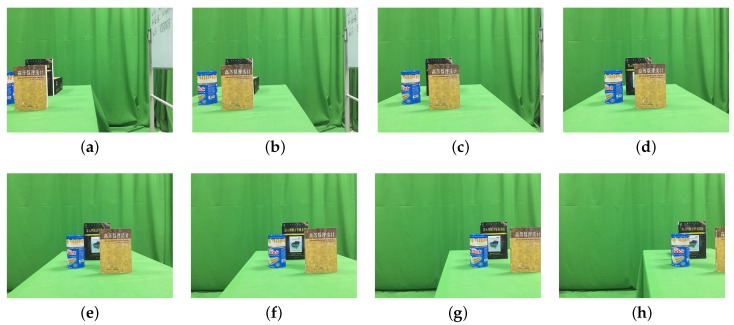
Examples of the input images in the second book scene. (**a**–**h**) are captured images by camera array in the same scene.

**Figure A2:**
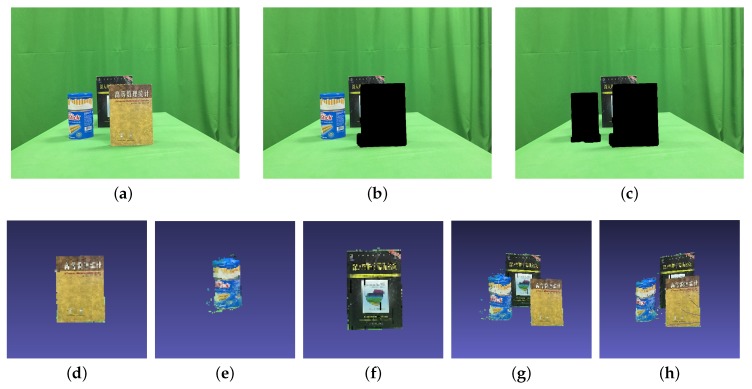
Reconstruction results of the can scene. (**a**) Reference camera view. (**b**) The front book removed. (**c**) Both the front book and the can removed. (**d**) Reconstructed front book. (**e**) Reconstructed can. (**f**) Reconstructed book behind. (**g**,**h**) Reconstructed scene in different slides.

In the following experimental evaluations, we captured indoor images by slide camera array, which is a virtual camera array of spatial and temporal sequences. There were eight viewpoints in this camera array, and image resolution was 960 × 720. We also made use of eight images as the input of our method. The focal length was 1170.1 measured in pixel units. This scene contains three objects, namely, the front book, the can, and the book behind. Please see Appendix A for more results. Different depths coexisted in this scene. Figure A1 shows the input images of the algorithm consisting of three steps in implementation. First, we focused on the front book, and labeled its pixels to obtain the images excluding the front-book region in each view (Figure 2b). Meanwhile, we reconstructed the front book as shown in Figure 2d. Second, we repeated the same procedure imposed on the can by performing labeling and pixel removal as shown in Figure 2c, and thus reconstructed this can as illustrated in Figure 2e. After removing the entire occluded region, we refocused on the book behind and reconstructed it according to Section 3.4. The reconstruction results of the three objects including can, the front book and the book behind accounted for 18,774, 46,075 and 32,696 points, respectively. Finally, after incorporating these point clouds into a single scene, we could derive the book scene as shown in Figure 2g,h.

Next, we moved our virtual-slide camera array outside to validate the effects of our method in a complex scene. To verify the capability of this method to eliminate occlusion, we captured both occlusion-free images and the ones with occlusion on the same target. As shown in Figure 3a, we could not capture a complete occlusion-free target in each camera view. After labeling and removing those occlusion pixels, the toy bear was reconstructed according to Section 3.4 (Figure 3c). Figure 3b shows SAI after removing the occlusion pixels. We also synthesized images that were captured with no occlusion (Figure 3e), and the toy bear without occlusion was reconstructed in Figure 3f. In this scene, the number of point clouds in the reconstruction results with and without occlusion conditions were 10,252 and 9679 respectively, which implies that our method can deal with occlusion conditions. To verify the robustness of our proposed method, we have collected massive amounts of data for 3D reconstruction.

Finally, we reconstructed objects with complex shapes, such as rocks, and discuss the problem of self-occlusion. In this scene, we used two pieces of rock that were placed in different positions to simulate self-occlusion in real scenes. Similarly, there were eight images used as input. The result of rock reconstruction is shown in Figure A6. Our method performed well in dealing with complex objects. The relative position between the two rocks is shown in Figure A6c,d. If the distance between the two rocks was too close, our method could not reconstruct the back rock well (please see Figure A6e,f).

## 5. Performance Evaluation and Discussion

**Figure A4:**
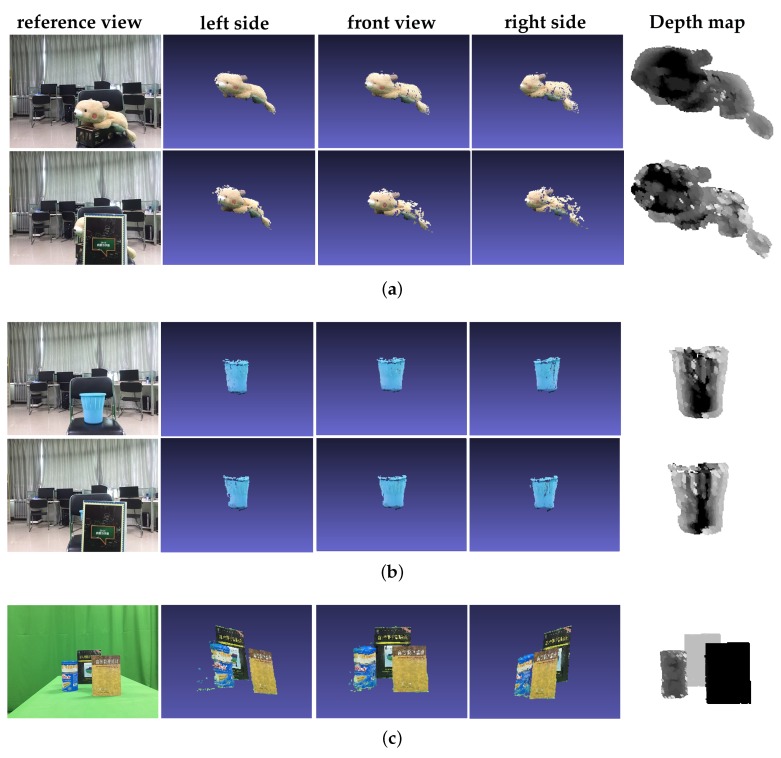
Indoor scene. (**a**) Toy-bear scene of reconstruction. (**b**) Trash-bin scene of reconstruction. (**c**) Book scene of reconstruction.

**Figure A5:**
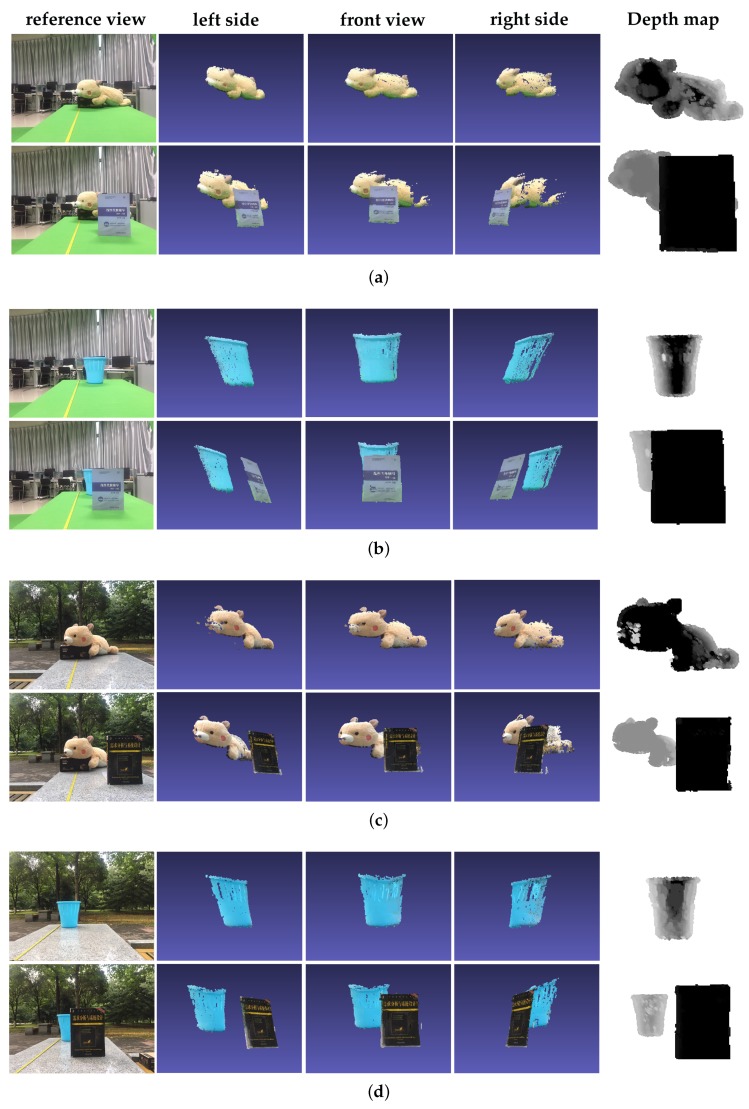
(**a**) Reconstructed toy bear in indoor scene. (**b**) Reconstructed trash bin in indoor scene. (**c**) Reconstructed toy bear in outdoor scene. (**d**) Reconstructed trash bin in outdoor scene.

**Figure A6:**
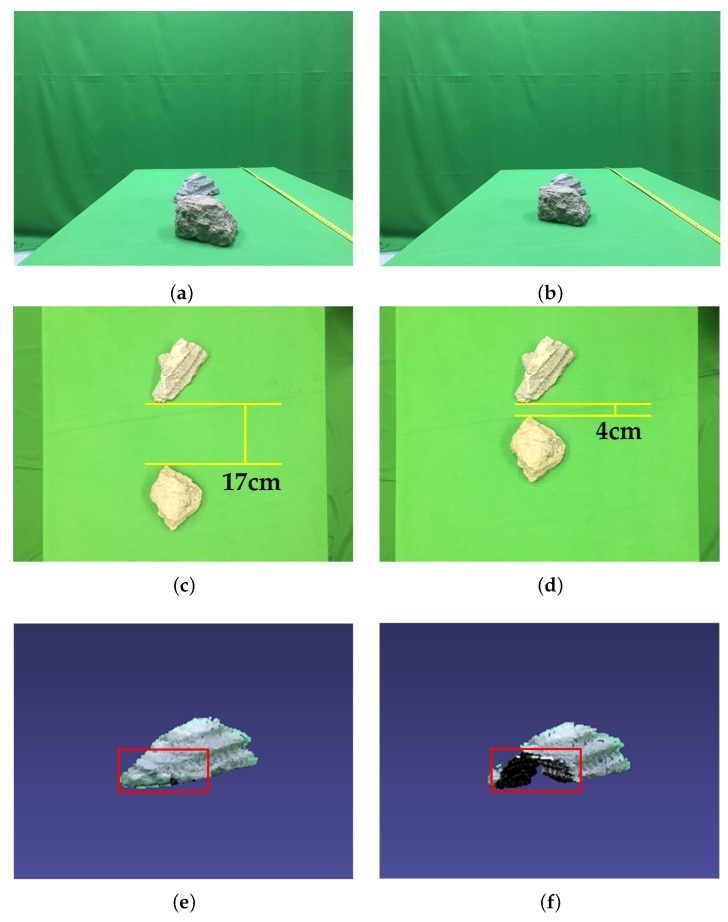
(**a**) Reference view of the rock scene, and the scenario of too-close and serious occlusion. (**b**) Reference view of the rock scene, and the scenario of general occlusion. (**c**,**d**) Top view of the rock scene. (**e**) Reconstruction result under serious occlusion condition. (**f**) Reconstruction result under general occlusion condition.

**Figure A7:**
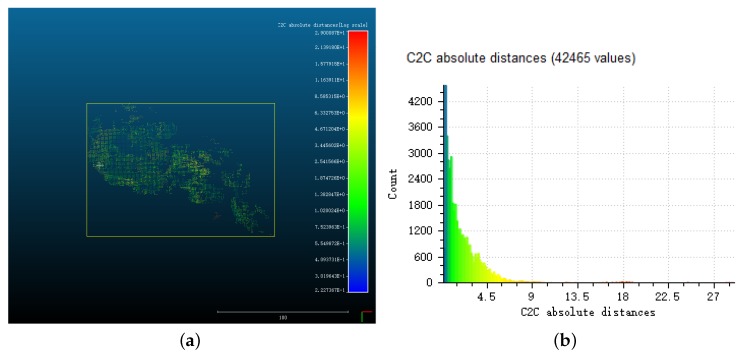
(**a**) Point cloud obtained by our method and graphical representation of spatial differences under occlusive and non-occlusive conditions. (**b**) Absolute distribution of point-cloud spatial differences.

Intuitively, we qualitatively evaluated the performance of our method. We separated an indoor scene from an outdoor scene, and reconstructed different objects with occlusion and non-occlusion, respectively. In the indoor scene, by comparing Figure A4a,b, it was observed that the shelter obscured a large area of the target object. Our method is also insensitive to object occlusion and achieved consistent reconstruction results. In this case, the occluded object could still be well reconstructed. In order to verify the difference between our method under occlusive and non-occlusive conditions, the reconstructed results are displayed in Figure A7. As we can see, most points match within 7.5 mm, and more than 42,000 points could be generated from only eight images captured by our camera array. As shown in Figure A9c, which is reconstructed by Patch-based Multiview Stereo (PMVS), most points match within 0.32 mm, yet there are 329 points in the reconstructed result. In another equally important outdoor scene, the proposed method still performed well, as shown in Figure A5. Observed from these results reported in Figure A4 and Figure A5, our method is robust and works well in complex scenes. Besides, it can also effectively reconstruct an occluded object under occlusion conditions.

**Figure A8:**
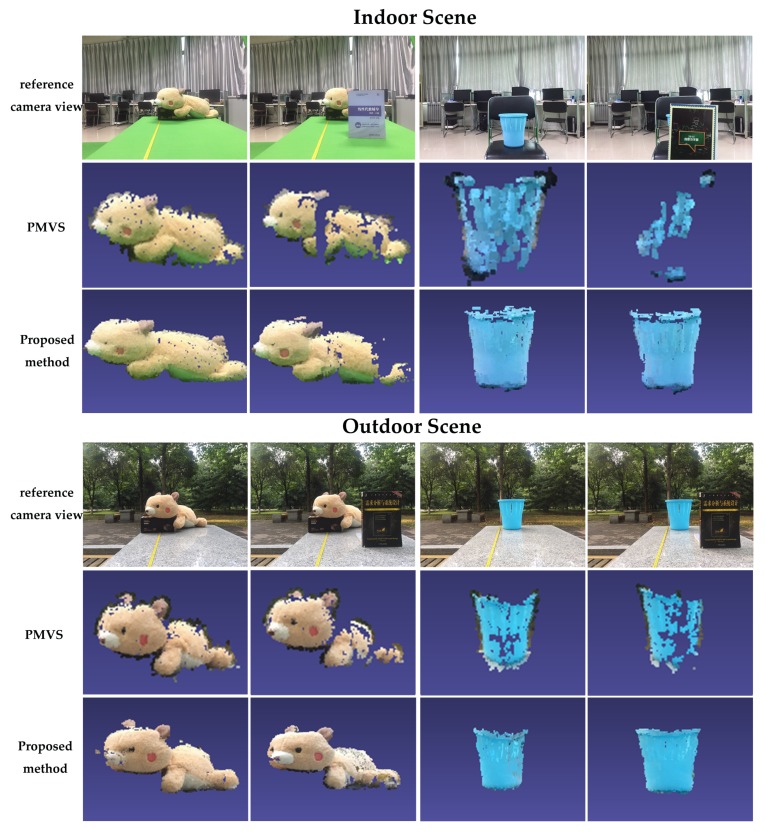
Comparison of reconstruction results achieved by different competing methods in different scenes with and without occlusion.

**Figure A9:**
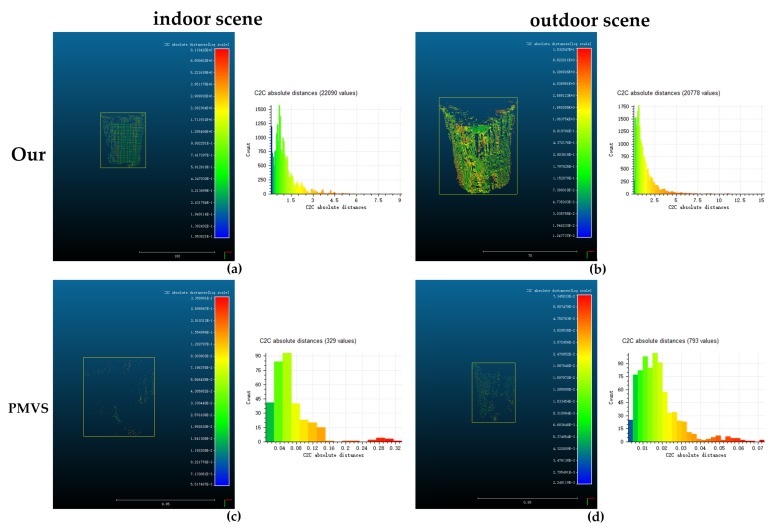
Comparison of PMVS and our method in the occluded trash bin scene shown in Figure A8. (**a**,**b**) Images obtained by our method in indoor and outdoor scenes, respectively. (**c**,**d**) Images obtained by PMVS in indoor and outdoor scenes, respectively.

**Figure A10:**
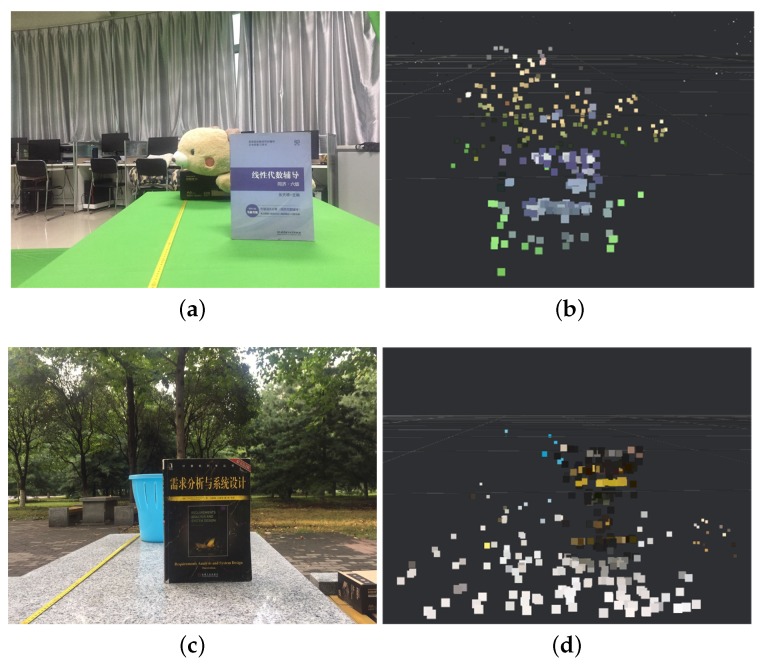
Illustrative reconstruction results achieved by MeshRoom software. Two different images in indoor and outdoor scenes are displayed on the left column (**a**,**c**), while the corresponding reconstruction results are shown as (**b**,**d**). It is observed that this method fails to reconstruct the objects in the scene.

Inspired by the research of 3D accuracy-assessment methods [42,43,44], we have compared the performance of our method with the PMVS method, which was included in the VisualSFM software. The corresponding reconstruction results are illustrated in Figure A8. In the software environment are conducted with Visual Studio 2017 which is made by Microsoft Corporation and open-source library OpenCV 3.4.0 on a Windows 10 operating system. Regarding the hardware environment, we made use of Core i5-4590 quad-core processor with 8G memory. For the indoor scene of toy bear with occlusion, our method takes about 93 s to get the point clouds while PMVS roughly 14 s. It is worth mentioning that our method may take more time, but our reconstruction result is much better than PMVS at the same time. In implementation, open-source software CloudCompare (version 2.10), which is created by the PhD of Daniel Girardeau-Montaut, was used to compare respective models of our method and VisualSFM software and it is a GUI application for 3D reconstruction using structure from motion (SFM) by wu changchang. As shown in Figure A9, the number of points reconstructed by our method was significantly higher than those obtained by the PMVS method in both indoor and outdoor scenes. However, the spatial differences of our method are higher than those of the PMVS method, which will be addressed in our future work. In addition, we attempted to use open-source software MeshRoom, which is based on Moulon’s method [45] and Jancosek’s method [46], to reconstruct the same scenes. Under the same conditions, their method demonstrated degraded performance and failed to achieve desirable object reconstruction results in some cases as shown in Figure A10.

Inspired by the comparison of image quality-assessment methods [47,48,49], Peak Signal-to-Noise Ratio (PSNR), Root Mean Squared Error (RMSE), Signal-to-Noise Ratio (SNR), and Mean Absolute Error (MAS) were used to quantitatively evaluate the reconstruction error of our method. Since the scale of different reconstruction results is inconsistent, we compared reconstruction results when occlusion was present and absent, respectively. Based on the comparison results, our method is more robust in response to 3D reconstruction tasks with occlusion (Table A1).

## 6. Conclusions

In this paper, we have proposed a novel method that uses camera array SAI to reconstruct an occluded object. The proposed method labels and removes occlusion pixels before refocusing on the occluded object to reconstruct it. The advantages of our method can be summarized as follows: First, the proposed method applies pixel labeling based on SAI to reconstruct objects, which implies that our approach could greatly reduce the impact of occlusion on reconstruction results. Second, these images are captured by camera arrays, and the positional relationship between cameras can be used to obtain high-precision results. Third, the camera array with a slide rail has greatly improved its flexibility to adapt to a more varying environment. Despite effective, our method still suffers from the following limitations: To distinguish object pixels from a visual background, it is difficult for the variance-based method to handle a background that has similar color with the object. As a result, of only one plane of the object being captured, the method cannot reconstruct a complete 3D model of the object. We will explore this problem in future work. In addition, developing a real-time occluded-object reconstruction system will be also included in our future work. 

## Figures and Tables

**Figure A3:**
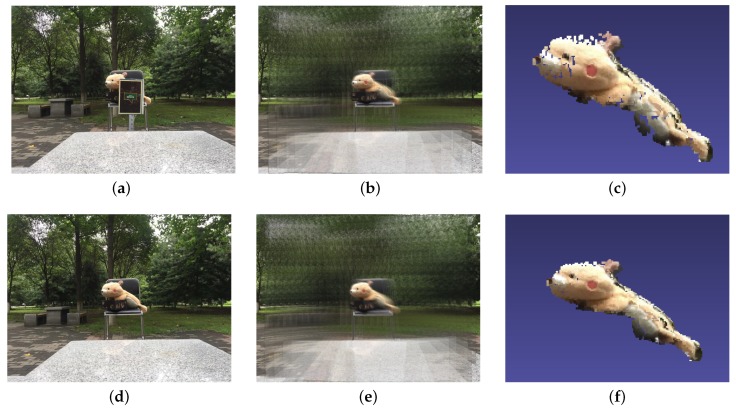
Outdoor scene. (**a**) Toy-bear scene with occlusion in reference camera view. (**b**) Synthetic aperture imaging with occlusion. (**c**) Reconstructed toy bear with occlusion. (**d**) Toy-bear scene without occlusion in reference camera view. (**e**) Synthetic aperture imaging without occlusion. (**f**) Reconstructed toy bear without occlusion.

## Appendix A

### Appendix A.1.

**Table A1:** Quantitative evaluations of the Patch-based Multiview Stereo (PMVS) method and our method.

Method	PSNR	SNR	RMSE	MAE
PMVS	17.56	9.54	34.30	4.47
Ours	23.84	11.4	18.76	0.83
